# Ca^2+^ Release Events in Cardiac Myocytes Up Close: Insights from Fast Confocal Imaging

**DOI:** 10.1371/journal.pone.0061525

**Published:** 2013-04-18

**Authors:** Vyacheslav M. Shkryl, Lothar A. Blatter

**Affiliations:** 1 Deptartment of General Physiology of the Nervous System, A. A. Bogomoletz Institute of Physiology, Kiev, Ukraine; 2 Department of Molecular Biophysics and Physiology, Rush University Medical Center, Chicago, Illinois, United States of America; University of Queensland, Australia

## Abstract

The spatio-temporal properties of Ca^2+^ transients during excitation-contraction coupling and elementary Ca^2+^ release events (Ca^2+^ sparks) were studied in atrial and ventricular myocytes with ultra-fast confocal microscopy using a Zeiss LSM 5 LIVE system that allows sampling rates of up to 60 kHz. Ca^2+^ sparks which originated from subsarcolemmal junctional sarcoplasmic reticulum (j-SR) release sites in atrial myocytes were anisotropic and elongated in the longitudinal direction of the cell. Ca^2+^ sparks in atrial cells originating from non-junctional SR and in ventricular myocytes were symmetrical. Ca^2+^ spark recording in line scan mode at 40,000 lines/s uncovered step-like increases of [Ca^2+^]_i_. 2-D imaging of Ca^2+^ transients revealed an asynchronous activation of release sites and allowed the sequential recording of Ca^2+^ entry through surface membrane Ca^2+^ channels and subsequent activation of Ca^2+^-induced Ca^2+^ release. With a latency of 2.5 ms after application of an electrical stimulus, Ca^2+^ entry could be detected that was followed by SR Ca^2+^ release after an additional 3 ms delay. Maximum Ca^2+^ release was observed 4 ms after the beginning of release. The timing of Ca^2+^ entry and release was confirmed by simultaneous [Ca^2+^]_i_ and membrane current measurements using the whole cell voltage-clamp technique. In atrial cells activation of discrete individual release sites of the j-SR led to spatially restricted Ca^2+^ release events that fused into a peripheral ring of elevated [Ca^2+^]_i_ that subsequently propagated in a wave-like fashion towards the center of the cell. In ventricular myocytes asynchronous Ca^2+^ release signals from discrete sites with no preferential subcellular location preceded the whole-cell Ca^2+^ transient. In summary, ultra-fast confocal imaging allows investigation of Ca^2+^ signals with a time resolution similar to patch clamp technique, however in a less invasive fashion.

## Introduction

Excitation-contraction coupling (ECC) refers to the process that links cell membrane depolarization to Ca^2+^ mobilization and development of contractile force in the heart [1]. Action potential (AP) depolarization activates voltage-gated L-type Ca^2+^ channels (LCC; also referred to as dihydropyridine receptors) in the surface membrane, resulting in localized (sub-membrane) increases in cytosolic Ca^2+^ ([Ca^2+^]_i_). Calcium that enters the cell activates sarcoplasmic reticulum (SR) Ca^2+^ release channels (ryanodine receptors, RyRs) through a process known as calcium-induced calcium release (CICR; [2]). RyRs and LCCs face each other in the diadic cleft formed by the close approximation of surface and SR membranes. RyRs assemble as clusters of channels in the SR membrane and form individual Ca^2+^ release units (CRU; [3]). Together with the diadic cleft and sarcolemmal Ca^2+^ channels CRUs are organized in discrete signaling domains or couplons [4]. In response to an AP hundreds of CRUs are activated essentially simultaneously. The spatial and temporal summation of Ca^2+^ release from individual CRUs gives rise to the whole-cell Ca^2+^ transient [5], [6], [7]. Because of the extensive transverse (t) tubular network - a 3-dimensional network of surface membrane invaginations that assure physical proximity of surface membrane Ca^2+^ channels and CRUs throughout the entire cell volume [8] - AP-induced Ca^2+^ release in ventricular myocytes is highly synchronized and spatially rather homogeneous.

In contrast, in atrial cells the t-tubular system is poorly developed or even entirely lacking [7], [9], [10], [11], [12]. In atrial cells two types of SR can be defined, based on their location relative to the surface membrane. Junctional SR (j-SR) is found in the cell periphery where it is organized in peripheral couplings, i.e. the SR membrane is found in close spatial association with the surface membrane, similar to the diadic cleft in ventricular myocytes [13], [14]. In contrast, non-junctional SR (nj-SR) is found in deeper regions of the cell and does not associate with the surface membrane. Both j-SR and nj-SR possess RyRs [10], [11], [12], [13], [15], [16], [17] which are organized in a 3-dimensional array of RyR clusters, and are capable of active SR Ca^2+^ release. As a consequence of these ultrastructural arrangements AP-induced Ca^2+^ release is spatially inhomogeneous (for review see e.g. [7], [18], [19]): AP-induced membrane depolarization activates Ca^2+^ entry through LCCs and CICR through RyRs of the j-SR. Elevation of peripheral [Ca^2+^]_i_ propagates via CICR in a Ca^2+^ wave-like fashion in centripetal direction by a diffusion-reaction process or a ‘fire-diffuse-fire’ mechanism [20].

Elementary Ca^2+^ release events, arising from the activation of individual CRUs, have been termed Ca^2+^ sparks ([21]; for a recent comprehensive review see [22]), although Ca^2+^ release events of smaller magnitude, possibly arising from single RyRs, have been postulated [23], [24], [25]. To date Ca^2+^ sparks have been detected in a wide range of mammalian species (for review see [22]). In atrial cells Ca^2+^ sparks originate from j-SR and nj-SR, however with a high prevalence for the cell periphery [7], [16], [26], [27], [28].

While sparks have been demonstrated in cardiac myocytes already two decades ago [21], it has remained a matter of debate, how many RyRs within a CRU contribute to a spark. Available data on how many RyRs a CRU of a cardiac myocyte contains vary widely and range from less than ten to several hundred RyRs [29], [30], [31], [32], [33], [34], [35], [36]. However, it is still not established whether a fraction or all RyRs of a cluster are activated in a spark. Indeed, published reports on how many channels contribute to a cardiac spark vary widely and range from a single or only a few channels to the whole CRU, thus possibly involving several hundred channels [21], [32], [37], [38], [39], [40], [41], [42].

The fact that such critical questions have remained inconclusive is, at least in part, linked to methodological and technical limitations hampering the recording of locally restricted and rapidly changing subcellular Ca^2+^ signals. The last two decades have seen significant improvements in Ca^2+^ indicator dyes that have allowed the field to move forward at considerable pace [43]. Nonetheless, the vast majority of studies on local Ca^2+^ signaling have used line scan confocal microscopy in conjunction with fluorescent Ca^2+^ indicator dyes. While line scan imaging provides a reasonable temporal resolution (typical sampling rates used are in the 0.5–1 kHz range) it is severely limited by the fact that a cellular event such as a Ca^2+^ spark, a Ca^2+^ wave or the whole-cell Ca^2+^ transient occurs in a 3-dimensional space, however its optical recording by line scan imaging is reduced to one spatial dimension. Therefore, line scan data notoriously suffer from out-of-focus problems (the event of interest originates from a different optical plane than the recording plane) and how reliably complex 3-dimensional spatial properties can be captured with this approach. Recently, novel technical imaging tools, consisting of a fast 2-D (or x-y) confocal scanning mechanism (LSM 5 LIVE slit scanner) in conjunction with the ability to move the plane of focus vertically in rapid and reproducible manner, have enabled us to achieve x-y-z-t (or 4-D) imaging of sparks with a reasonable time resolution und to gain novel insight into the properties of cardiac Ca^2+^ sparks [41]. With this technique we were able to determine the true in-focus amplitude of Ca^2+^ sparks and make the observation that sparks had a modal amplitude distribution. Here, we extended fast confocal imaging to the study of AP-induced Ca^2+^ transients in atrial and ventricular myocytes with the goal to gain new insight into Ca^2+^ entry, local Ca^2+^ release and cellular spread of activation during ECC, and to explore Ca^2+^ sparks at high temporal resolution to reveal the characteristics of the underlying Ca^2+^ release and gating properties of the channels involved.

## Materials and Methods

### Myocyte Isolation

Single myocytes were isolated from cat [26], [44], [45], [46] and rabbit [47] hearts as described previously. The investigation conforms with the Guide for the Care and Use of Laboratory Animals of the National Institutes of Health. All procedures and protocols for animal handling and cell isolation were fully approved by the Institutional Animal Care and Use Committee of Loyola University Chicago (Permit number 05-016) and the Institutional Animal Care and Use Committee of Rush University Medical Center Chicago (Permit number 09-055). All efforts were made to minimize suffering. Briefly, animals were anesthetized with thiopental sodium (50 mg kg^−1^, I.P.). After thoracotomy, hearts were excised, mounted on a Langendorff apparatus, and retrogradely perfused via the aorta with oxygenated collagenase (cats) or Liberase Blendzyme TH (rabbits) containing solution (37°C). Myocytes were used for experimentation within 1–6 hours after isolation.

### Chemicals, Solutions and Experimental Conditions

Chemicals were obtained from Sigma-Aldrich (St. Louis, MO) unless otherwise noted. During experiments myocytes were superfused continuously with normal Tyrode solution (composition in mM: NaCl 135: KCl 4; CaCl_2_ 2; MgCl_2_ 1; D-glucose 10; HEPES 10; pH 7.4 adjusted with NaOH). All experiments were performed at room temperature (22–25°C). APs were elicited by electrical field stimulation by applying 1 ms voltage pulses of suprathreshold amplitude through a pair of platinum electrodes.

### [Ca^2+^]_i_ Measurements and Confocal Microscopy

Changes of [Ca^2+^]_i_ were measured using fluorescence laser scanning confocal microscopy. Intact atrial and ventricular myocytes were loaded with the fluorescent Ca^2+^ indicator Fluo-4 by 20 min incubation in Tyrode solution containing 10 µM of the membrane permeant acetoxymethyl ester form of the indicator (Fluo-4/AM; Molecular Molecular Probes/Life Technologies, Grand Island, NY) at room temperature. 15–20 min were allowed for de-esterification of the dye. Fluo-4 was excited with the 488 nm line of an argon ion laser and emitted fluorescence was measured at wavelengths >515 nm. Confocal imaging at high temporal resolution was performed with a slit scanning confocal microscope (LSM 5 LIVE; Carl Zeiss, Oberkochen, Germany) equipped with a 63×, 1.20 n.a. water-immersion objective (C-Apochromat; Carl Zeiss). High-speed data acquisition was achieved by bi-directional scanning. 2-dimensional (2-D) imaging (also referred to as x-y imaging) yielded images of 512×53 or 512×31 pixels (pixel distance of 0.3 µm) recorded at 1038 and 1719 Hz, respectively. The x-dimension is defined as the longitudinal axis of the cell. The y-dimension refers to the transverse cell axis and the z-dimension defines the axial (or vertical) cell dimension. The point-spread function of the system had full-width at half maximum (FWHM) values of 0.52, 0.46, and 1.25 µm, respectively, in the x, y, and z directions [41]. Changes of [Ca^2+^]_i_ are presented as background-subtracted normalized fluorescence (ΔF/F_0_) where F is the fluorescence intensity, F_0_ is resting fluorescence recorded under steady-state conditions at the beginning of an experiment, and ΔF = F–F_0_. Average data are presented as mean ± SEM.

### Electrophysiology

Experiments for simultaneous [Ca^2+^]_i_ and membrane Ca^2+^ current measurements were performed in rabbit ventricular myocytes using the voltage-clamp technique in the whole-cell ruptured patch configuration, using an Axopatch 200A amplifier, the Axon Digidata 1440A interface and pCLAMP 10.2 software (Molecular Devices, Sunnyvale, CA). The patch pipette solution contained (in mM): 120 L-aspartate; 120 CsOH; 20 TEA-Cl; 20 HEPES; 1 L-gluthathione (reduced); 1 DM-nitrophen (EMD Chemicals, Philadelphia, PA); 4 ATP-Na, 0.25 CaCl_2_ and 0.1 Fluo-4 pentapotassium salt (Molecular Probes); pH 7.2 adjusted with CsOH). Currents were recorded at 100 kHz and low-pass filtered at 5 kHz.

## Results

### Spatio-temporal Characteristics of Ca^2+^ Transients and Ca^2+^ Sparks in Atrial Myocytes

**Figure 1:**
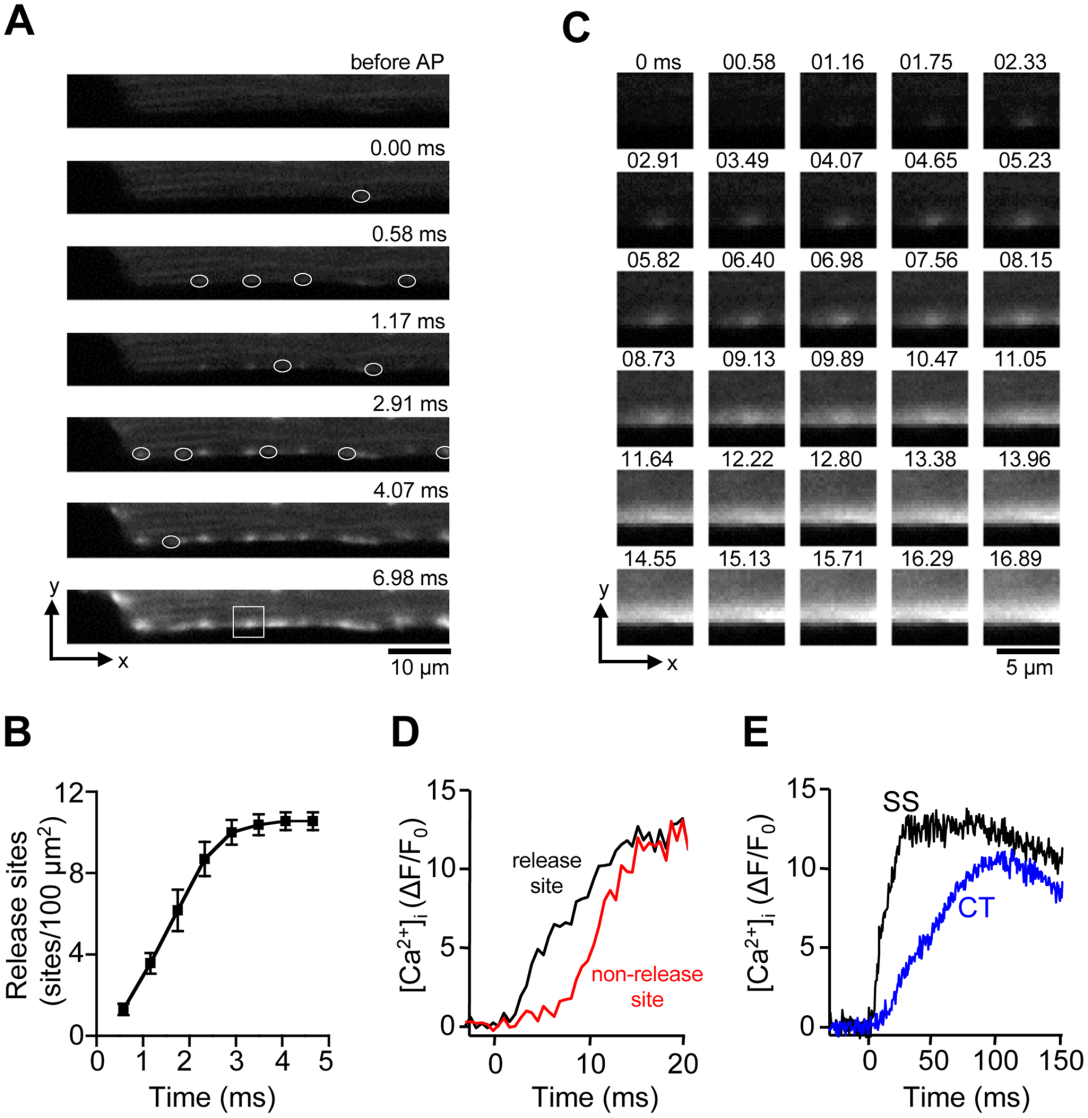
AP-induced Ca^2+^ transients in atrial myocytes. A, series of confocal 2-D images of the early phase of a Ca^2+^ transient evoked by electrical field stimulation. White ovals mark the first appearance of individual Ca^2+^ release sites of the j-SR in the SS region. t = 0 indicates the image frame where the first activated CRU was detected. **B**, cumulative recruitment of individual j-SR CRUs. CRUs were detected in a SS region of 75 µm×2 µm and normalized to number of CRUs/100 µm^2^. **C**, Sequential 2-D images of an individual SS j-SR release site, marked by the white box in panel A, recorded at 0.58 ms time intervals. **D**, Evolution of [Ca^2+^]_i_ at an individual SS CRU and an adjacent non-release site at a distance of ∼1 µm. The traces were recorded from a 1 pixel sized regions (0.09 µm^2^). **E**, comparison of SS (j-SR) and CT (nj-SR; recorded at a distance of ∼4 µm from the cell membrane) Ca^2+^ transients.

In a first set of experiments we investigated the spatio-temporal organization of Ca^2+^ transients during normal ECC in atrial myocytes. AP-dependent Ca^2+^ transients were elicited by electrical field stimulation (0.7 Hz). As shown previously cat atrial myocytes are devoid of a t-tubular network [7], however RyR Ca^2+^ release channels are present throughout the cell with the exception of the nuclear region [13]. Fig. 1A shows a sequence of 2-D [Ca^2+^]_i_ images of the early phase of an electrically evoked Ca^2+^ transient in an atrial cell, recorded at 1719 Hz from a central axial plan, i.e. at equal distance from the top and bottom cell borders in the axial (z) dimension. Ca^2+^ release appeared spatially inhomogeneous and initiated at discrete sites in the periphery where the j-SR is located. Ca^2+^ release was also found to be asynchronous among peripheral j-SR release sites and required 3.0±0.1 ms (n = 7 atrial myocytes) for all detectable peripheral release sites to be activated (Fig. 1B). This is likely an underestimation of the time required to recruit all j-SR release sites because the appearance of new sites at later times becomes increasingly obscured by the overall increase of [Ca^2+^]_i_. Fig. 1C shows a single peripheral release site at higher magnification. The series of 2-D images reveals that from the point source of Ca^2+^ of the release site, Ca^2+^ diffuses laterally (x-dimension), and also centripetally (y-dimension). Fig. 1D shows the time course of the increase of [Ca^2+^]_i_ at an individual j-SR release site and a peripheral subsarcolemmal (SS) non-release site at a lateral distance of ∼1 µm, revealing a time delay that was characteristic for subsarcolemmal Ca^2+^ diffusion. Traces representing the SS j-SR region were recorded within <1 µm from the edge of the cell. Ca^2+^ release from peripheral j-SR sites eventually gave rise to a rather homogeneous elevation of [Ca^2+^]_i_ in the cell periphery, obscuring individual release sites. On average this homogeneous ‘ring’ of increased [Ca^2+^]_i_ was complete 10.1±1.5 ms after application of the electrical stimulus. As shown in Fig. 1E [Ca^2+^]_i_ started to rise with a delay at a central (CT) subcellular location distant from the cell membrane. At this location, which corresponds to the nj-SR, [Ca^2+^]_i_ rose much more slowly and peaked at a level that was below peak [Ca^2+^]_i_ in the cell periphery. As previously established [13], [26] activation of Ca^2+^ release from release units of the nj-SR in atrial cells occurs by propagating CICR or a ‘diffuse-fire-diffuse’ mechanism where Ca^2+^ released from a peripheral release site diffuses over a short distance (1–2 µm) to a neighboring more centrally located release site where it activates CICR. Through this sequence of events activation of Ca^2+^ release in atrial cells becomes spatially inhomogeneous and propagates from the periphery to the center of the myocyte in a Ca^2+^ wave-like fashion.

**Figure 2:**
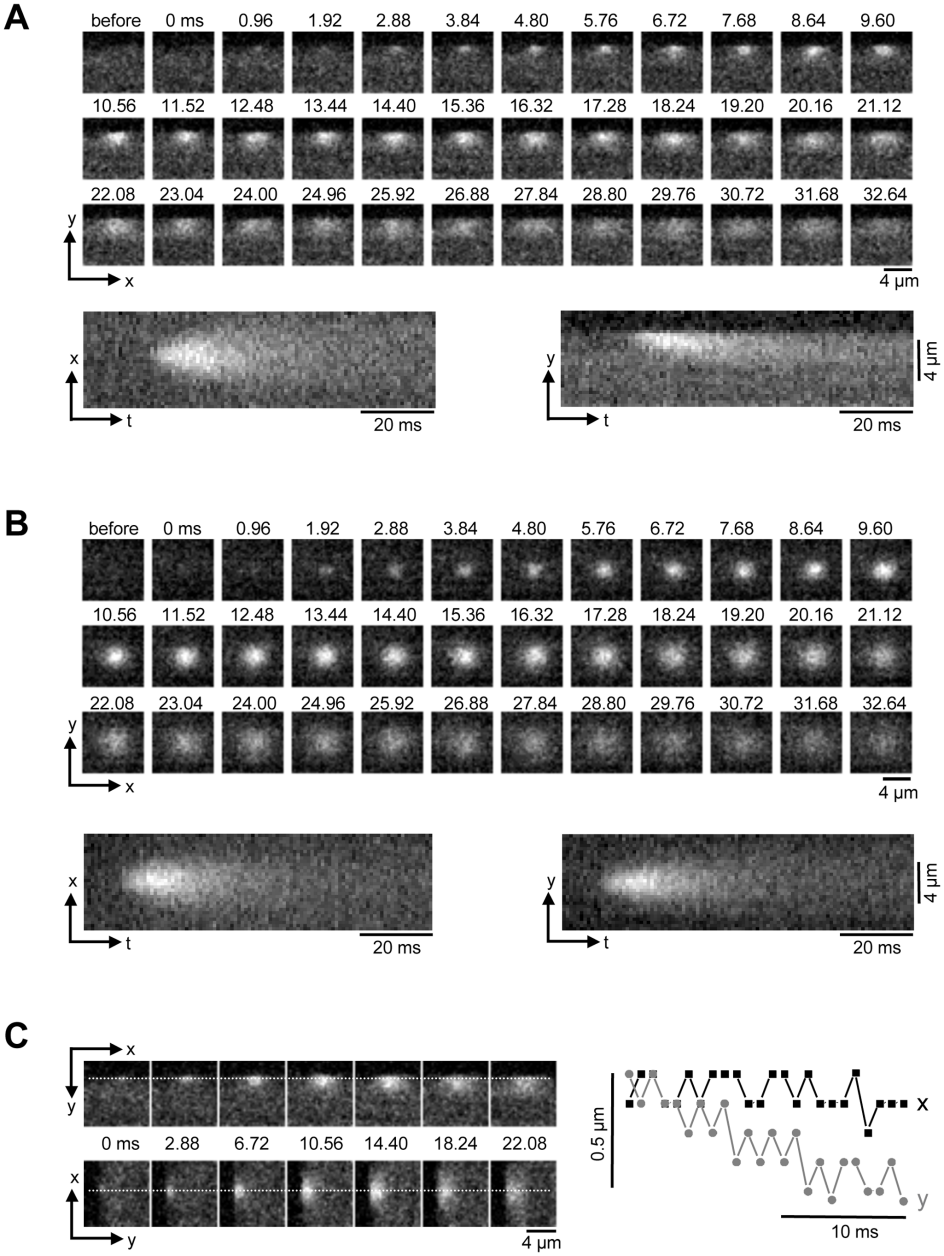
Ca^2+^ sparks in atrial myocytes. A, Series of confocal 2-D images of a spontaneous Ca^2+^ spark originating from the SS j-SR, recorded at 0.96 ms intervals (top). Bottom: reconstructed line scan images (x-t and y-t) from the 2-D series. **B**, analogous to panel A: spontaneous Ca^2+^ spark recorded from a CT nj-SR Ca^2+^ release unit. **C**, movement of the x-y -coordinates of maximal fluorescence during a SS Ca^2+^ spark. Left: the spatial coordinates of peak fluorescence intensity were recorded along the dashed lines in x- and y-dimension. Right: x-t and y-t plots of the location of maximal fluorescence, indicating a preferential movement of the point source of Ca generating the spark in centripetal direction.

It is generally agreed that the AP-induced whole-cell Ca^2+^ transient is the result of spatial and temporal summation of elementary Ca^2+^ release events from individual CRU, termed Ca^2+^ sparks [21], [22]. To gain additional insight into the process that initiates the Ca^2+^ transient in atrial cells we investigated spontaneous Ca^2+^ sparks from peripheral j-SR CRUs. Fig. 2A shows a series of 2-D images of a spontaneous peripheral Ca^2+^ spark. Images were recorded at a sampling rate of 1038 Hz. Sparks in the cell periphery arising from atrial j-SR CRUs are highly anisotropic with a preferential spread in the longitudinal direction. FWHM in longitudinal (x-dimension) direction was 2.50±0.07 µm (n = 30 sparks analyzed), whereas the average FWHM in transverse direction was 1.51±0.05 µm. Below the panel of 2-D images, two line scan images (x-t- and y-t-images) are shown that were constructed from the stack of 2-D images. For the x-t image a single line of pixels in the x-dimension was extracted at a fixed value of y from each consecutive 2-D image and the lines were stacked from left to right along the time axis. Analogously, a single line of pixels in the y-dimension was extracted at a fixed value for x. While the x-t-image shows a symmetrical spread of [Ca^2+^]_i_ from the site of initiation, the y-t-image clearly shows an anisotropic spread. The degree of anisotropy was quantified as eccentricity (e). Eccentricity was defined as the ratio of FWHM of Ca^2+^ sparks measured along two perpendicular axis within the focal plane and by convention the larger value was divided by the smaller measurement, or e = FWHM_wide_/FWHM_narrow_. For peripheral j-SR Ca^2+^ sparks in atrial myocytes the average eccentricity was e = 1.71±0.05, i.e. peripheral sparks were approximately 70% wider along the longitudinal cell axis with all sparks analyzed extending more in the longitudinal dimension.

In contrast, sparks recorded from central (nj-SR) regions revealed a different geometry (Fig. 2B). Sparks were essentially symmetrical in x and y, with an average eccentricity of e = 1.12±0.03, i.e. on average FWHM measured along two perpendicular axes differed by 12% only with no preferential direction.

The structural arrangements of surface cell and SR membranes in the peripheral couplings of the j-SR can explain the preferential spread of Ca^2+^ when imaged in a single confocal plane cutting across such a coupling. As discussed above, during ECC Ca^2+^ release propagates by CICR from the cell periphery to the center of the cell. We therefore explored the spatio-temporal properties of peripheral Ca^2+^ sparks for patterns that indicate transverse or centripetal spread of Ca^2+^. For this purpose we determined frame-by-frame the x-y-coordinates of maximal fluorescence which identifies a point source of Ca^2+^ release. As shown in Fig. 2C the location of maximal fluorescence was stable in the longitudinal (x) direction, arguing for a symmetrical lateral dissipation of Ca^2+^ out of the diadic cleft of the peripheral couplings. In contrast, in transverse (y) direction the location of maximal fluorescence shifted by ∼0.5 µm from the cell membrane towards cell center over the time course of 20 ms, thus facilitating the spread of excitation in centripetal direction. This spatial pattern cannot be explained by simple Ca^2+^ diffusion away from a fixed point source of Ca^2+^ and may suggest a preferential direction of Ca^2+^ release propagation possibly within a peripheral CRU.

### Spatio-temporal Characteristics of AP-induced Ca^2+^ Transients in Ventricular Myocytes

Ventricular myocytes are equipped with an extensive t-tubular membrane system that is organized in a sarcomeric pattern and functionally allows conduction of the AP into deeper regions of the myocyte. Transverse tubules provide the structural basis that CRUs are all in close contact with the cell membrane that hosts voltage-gated Ca^2+^ channels. Through this arrangement membrane depolarization by an AP allows the synchronous delivery of a triggering amount of Ca^2+^ into the diadic cleft (formed by t-tubular and SR membranes) to induce CICR. Because the t-tubular system guarantees synchronicity for CICR activation, the onset of Ca^2+^ release appears spatially and temporally rather homogeneous, although upon closer examination inhomogeneities have been observed [7], [26], [48], [49], [50].

**Figure 3:**
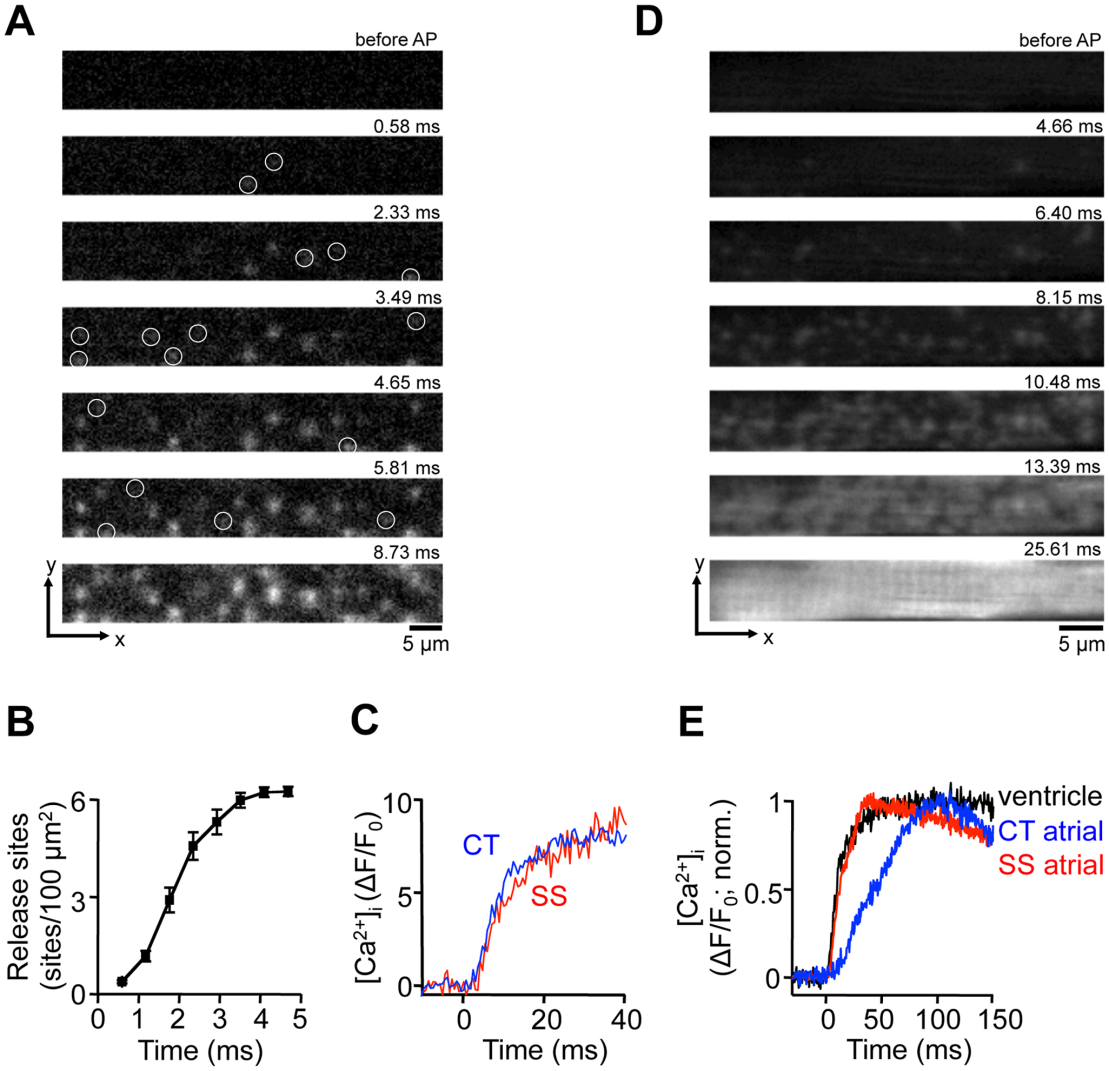
Ca^2+^ release signals in ventricular myocytes. A, series of confocal 2-D images of the early phase of a Ca^2+^ transient evoked by electrical field stimulation in a ventricular myocytes. White circles mark the first appearance of individual j-SR Ca^2+^ release sites. t = 0 indicates the image frame where the first activated CRU was detected. **B**, cumulative recruitment of individual j-SR CRUs. CRUs were detected in a region of interest encompassing the j-SR and measuring 60 µm×7.5 µm. Detected CRUs were normalized to number of CRUs/100 µm^2^. **C**, evolution of [Ca^2+^]_i_ at individual SS and CT j-SR release sites. The traces were recorded from a 1 pixel sized regions (0.09 µm^2^). **D**, series of confocal 2-D images of the early phase of a Ca^2+^ transient evoked by electrical field stimulation in an atrial myocyte and recorded from a focal plane positioned in the SS j-SR space at the bottom of the cell. **E**, normalized Ca^2+^ transients recorded from ventricular j-SR (black), CT nj-SR atrial (blue) and SS j-SR atrial (red) release sites.

Fig. 3A shows high-resolution 2-D images of the early phase of an AP-induced Ca^2+^ transient from a ventricular myocytes. The images were recorded from a central focal plane equidistant from the bottom and the top of the cell at a sampling rate of 1719 Hz. Several characteristic features of a ventricular Ca^2+^ transient become evident: (1) localized Ca^2+^ release signals from individual CRUs appeared randomly throughout the cell with no obvious preferential subcellular location; (2) recruitment of CRUs is not entirely synchronized. As shown in Fig. 3B the number of activated CRUs steadily increased over the course of ∼4 ms. On average, activation of all detectable release sites was reached after 3.9±0.1 ms (n = 9 ventricular myocytes). (3) Local increases of [Ca^2+^]_i_ from peripheral and central CRUs that were recruited at the same time, were essentially indistinguishable (Fig. 3C).

In atrial myocytes, the arrangement of surface membrane and j-SR membranes of the peripheral couplings have a certain resemblance with ventricular myocytes. The common features are that the peripheral couplings form diadic clefts were both membranes associate closely and voltage-gated Ca^2+^ channels face clusters of RyRs [13]. Peripheral couplings are found spaced approximately 2 µm apart. Therefore, we reasoned that the spatio-temporal pattern of CRU activation by an AP would be distinctly different from the one shown in Fig. 1A if we would be able to capture exclusively CRUs from the peripheral j-SR. For this purpose we positioned the focal plane at the bottom of the cell just above the bottom surface membrane. Fig. 3D shows a sequence of 2-D images of the recruitment of j-SR release sites induced by AP depolarization. Similar to ventricular myocytes, no preferential subcellular locations where activation started could be identified, recruitment of j-SR CRUs was temporally asynchronous and it required ∼10 ms for activation of all CRUs. Fig. 3E shows normalized local Ca^2+^ transients recorded from individual release sites of the atrial j-SR (SS), ventricular j-SR and atrial nj-SR (CT). The time course of the rapid rising phase of the ventricular and the atrial SS signals were virtually identical, whereas the rise of [Ca^2+^]_i_ at the atrial CT site was significantly slower. Thus, in respect to the dynamics of local Ca^2+^ release ventricular and atrial j-SR sites behaved remarkably similar, whereas release from atrial nj-SR CRUs revealed a distinctly different spatio-temporal pattern.

### Ultra-fast Line Scan Imaging of Ca^2+^ Sparks

It is generally agreed that Ca^2+^ sparks result from the concerted opening of a finite number of RyR Ca^2+^ release channels organized in clusters in the SR membrane. It is, however still a matter of debate how many individual release channels contribute to a spark signal, and the reported numbers vary widely, ranging from small numbers of less than a dozen to over a hundred such channels (see Introduction and Discussion).

**Figure 4:**
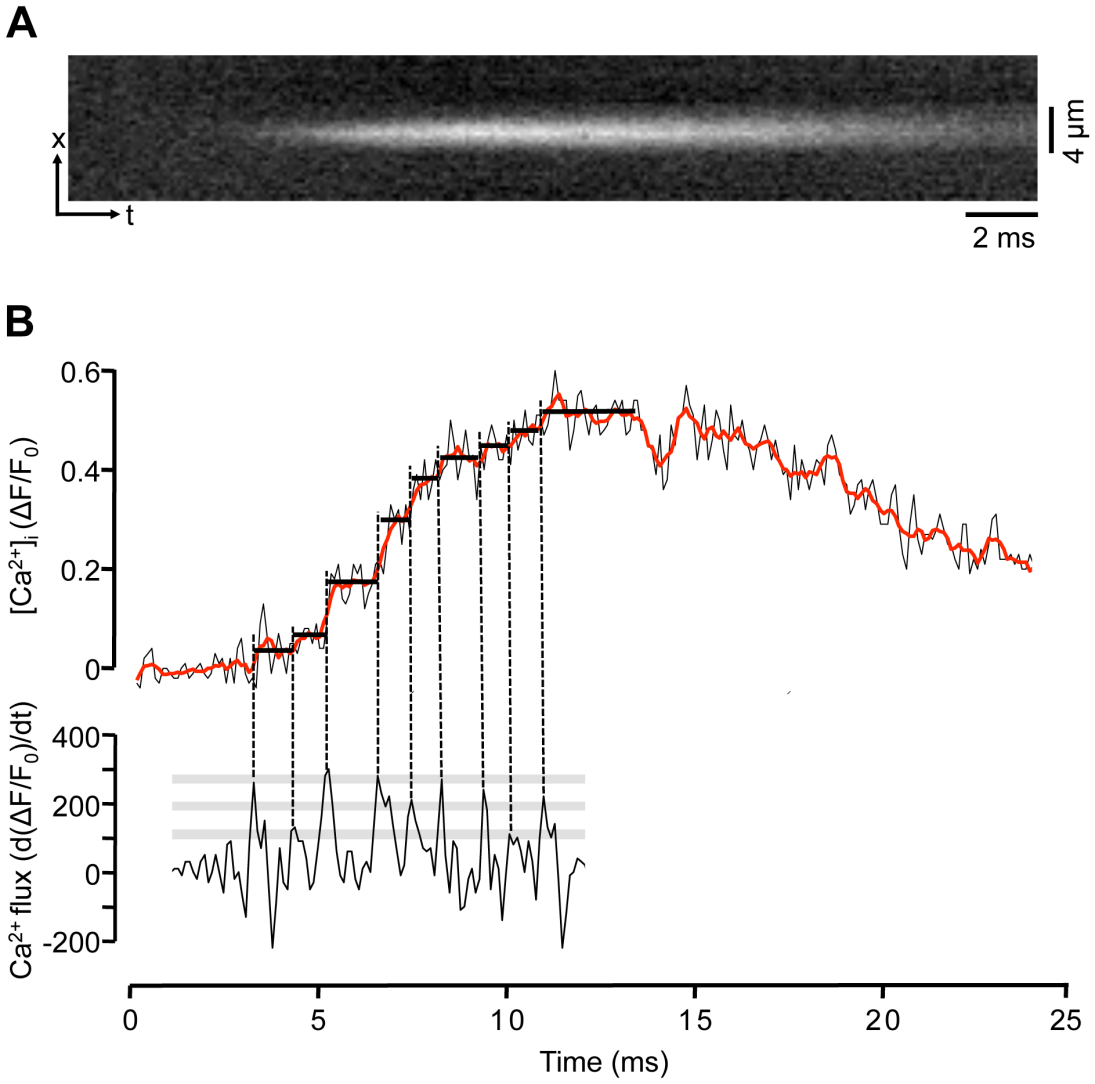
Ultra-fast Ca^2+^ spark recordings. A, x-t line scan image recorded at 40,000 lines/s. **B**, Top: one pixel-wide (0.3 µm) ΔF/F_0_ profile recorded from the center of the spark. For noise reduction data were averaged to 10,000 lines/s. The red trace represents a 5-point moving average. Bottom: d(ΔF/F_0_)/dt (s^−1^), first derivative of the ΔF/F_0_ signal of the rising phase of the spark. Vertical dashed lines mark maxima of the d(ΔF/F_0_)/dt signal identifying maxima of Ca^2+^ release flux. The discrete peaks of the d(ΔF/F_0_)/dt signal were used to identify step-like increases of the ΔF/F_0_ signal (marked by horizontal solid black lines). Grey bars indicate discrete d(ΔF/F_0_)/dt levels. A discrete d(ΔF/F_0_)/dt level was defined when at least two d(ΔF/F_0_)/dt peaks of the same amplitude were observed.

Conventional confocal line scan imaging of Ca^2+^ sparks typically employs scanning speeds in the 0.5–1 kHz range. Such time resolution can impose severe limitations for the fidelity of recording of fast components of a Ca^2+^ spark such as its rising phase, and may hamper the interpretation of such signals in terms of the underlying channel gating and number of channels involved. Taking advantage of the high temporal resolution capabilities of the LSM 5 LIVE, we recorded Ca^2+^ sparks in line scan (x-t) mode with an acquisition rate of 40 kHz (25 µs/line) as shown in Fig. 4A. Fig. 4B (top) shows a single pixel-wide (0.3 µm) fluorescence profile (ΔF/F_0_) obtained from the center of the spark. The spark shown had a rise time (time-to-peak) of ∼6 ms. Detailed analysis of the rising phase of the Ca^2+^ spark revealed step-like increases of [Ca^2+^]_i_ (marked by horizontal lines in Fig. 4B). To identify step-like changes of the rising phase of the spark the first derivative of the ΔF/F_0_ signal was calculated (d(ΔF/F_0_)/dt; Fig. 4B bottom). The first derivative of the ΔF/F_0_ signal represents an approximation of the underlying Ca^2+^ fluxes [26], [51]. During the rising phase of the spark the d(ΔF/F_0_)/dt revealed several discrete peaks identifying maxima of Ca^2+^ release flux. The timing of these peaks (vertical dashed lines) was used to identify discrete steps in the [Ca^2+^]_i_ signal marked by solid black lines in Fig. 4B. In the example shown (similar observation were made in 7 myocytes) 9 distinct levels of the ΔF/F_0_ signal could be identified with this approach. The improved temporal resolution of Ca^2+^ spark recording may open new avenues to investigate RyR gating within a CRU in-situ by a non-invasive approach as will be discussed below.

### Ca^2+^ Fluxes During ECC Identified with High-speed Imaging

**Figure 5:**
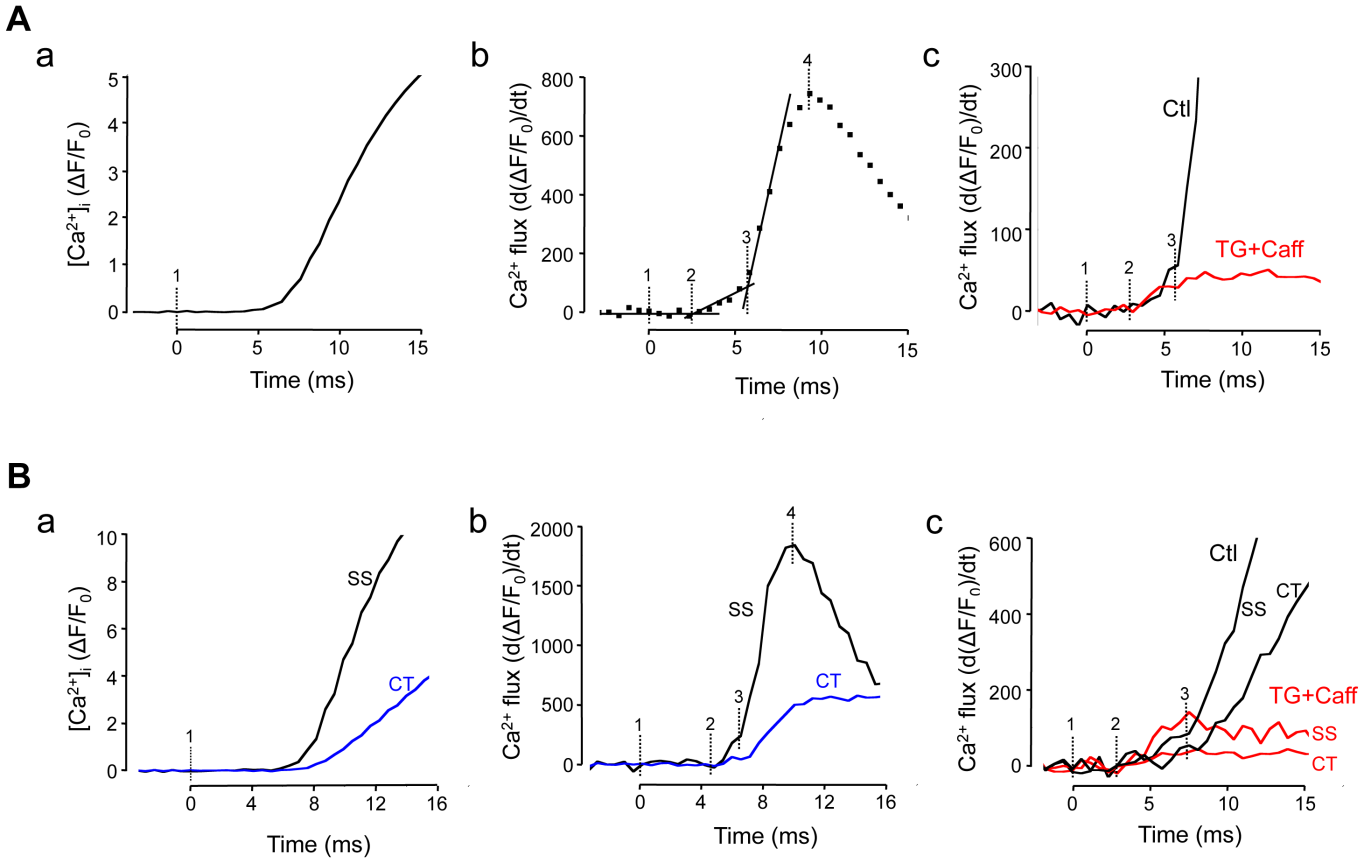
Subcellular Ca^2+^ fluxes during ECC identified by fast Ca^2+^ imaging. A, global Ca^2+^ transient (ΔF/F_0_) elicited by electrical field stimulation of a ventricular myocyte (panel **a**). Marker ‘1’ indicates the application of the electrical stimulus. Markers ‘2’ and ‘3’ were positioned according to the analysis in panel b. Panel **b**: first derivative of ΔF/F_0_ (s^−1^) from panel a, representing Ca^2+^ flux. Markers ‘2’ and ‘3’ indicate abrupt changes in Ca^2+^ flux rate, and marker ‘4’ indicates maximal Ca^2+^ flux. Panel **c**: spatially averaged Ca^2+^ flux under control conditions (Ctl) and during inhibition of SR function with thapsigargin (TG; 1 µM) and caffeine (Caff; 10 mM). **B**, subcellular Ca^2+^ transients (ΔF/F_0_) elicited by electrical field stimulation and recorded from subsarcolemmal (SS) j-SR and central (CT) nj-SR regions of an atrial myocyte (panel **a**). Panel **b**: first derivative of SS and CT Ca^2+^ signals representing subcellular Ca^2+^ flux rates. Markers ‘1’ to ‘4’ as in panel A. Panel **c**: subcellular (SS, CT) Ca^2+^ flux rates in control and in the presence of thapsigargin+caffeine. 2-D images were recorded 1719 Hz time resolution.

During ECC AP depolarization enables Ca^2+^ entry through voltage-gated Ca^2+^ channels that is followed by CICR from the SR. Thus, the global Ca^2+^ transient is the result of Ca^2+^ flux across the cell membrane and a much larger Ca^2+^ flux through RyR SR Ca^2+^ release channels. In the next set of experiments we set out to identify these two flux components with high speed Ca^2+^ imaging. Fig. 5Aa shows a whole cell global Ca^2+^ transient (ΔF/F_0_) induced by an AP in a ventricular cell and averaged over a region of interest of ∼550 µm^2^ (200×31 pixels) to obtain a low noise signal. Marker ‘1’ identifies the time when the electrical stimulus was applied to trigger an AP. Fig. 5Ab shows the first derivative of the [Ca^2+^]_i_ signal (d(ΔF/F_0_)/dt) representing the underlying Ca^2+^ flux. The first derivative signal revealed three distinct phases. The first phase, defined by the time of application of the electrical stimulus (marker ‘1’) and the begin of the rise of the d(ΔF/F_0_)/dt signal (marker ‘2’) was defined as the latency period between electrical stimulus and activation of Ca^2+^ flux, presumably resulting from Ca^2+^ entry. The latency period was 2.5±0.3 ms (n = 9 ventricular myocytes). During the following phase (between markers ‘2’ and ‘3’) d(ΔF/F_0_)/dt increased steadily at a slow rate. At t = 5.5±0.2 ms (marker ‘3’) the flux rate rather abruptly increased and peaked (marker ‘4’) on average at d(ΔF/F_0_)/dt = 748±71 s^−1^, 9.9±0.7 ms after application of the electrical stimulus. Fig. 5Ac compares the Ca^2+^ fluxes in control ventricular cells and myocytes with a disabled SR. The SR was disabled by complete depletion with caffeine (10 mM) and prevention of refilling by treatment with the SERCA blocker thapsigargin (1 µM). Elimination of SR Ca^2+^ release abolished the large increase of Ca^2+^ flux normally observed after marker ‘3’, however the latency period (2.1±0.1 ms; n = 11 myocytes) and the subsequent slow rise of the d(ΔF/F_0_)/dt signal were preserved. The d(ΔF/F_0_)/dt signal peaked at 50±5 s^−1^, 7.7±0.7 ms after the electrical stimulus. In conclusion, these observations are consistent with the hypothesis that phase ‘1–2’ (latency) marks the time interval that is required for the spread of depolarization and activation of surface membrane ion channels. Phase ‘2–3’ represents Ca^2+^ entry flux, and was identical in control myocytes and cells with disabled SR function. Phase ‘3–4’ is identified as Ca^2+^ release flux from the SR, since it was completely abolished when the SR was eliminated.

Fig. 5B shows an analogous analysis of data obtained in atrial myocytes. In atrial cells Ca^2+^ fluxes were further analyzed separately for SS (j-SR) and CT (nj-SR) regions. Fig. 5Ba shows the time course of the increase of [Ca^2+^]_i_ (ΔF/F_0_) induced by electrical field stimulation applied at marker ‘1’, revealing the delayed and slower rise of [Ca^2+^]_i_ in the CT region. Fig. 5Bb shows the first derivative of the ΔF/F_0_ representing the underlying Ca^2+^ fluxes. In the SS region, similar to ventricular cells, a latency period (3.6±0.7 ms; n = 6 atrial myocytes), was followed by a slow increase (starting at marker ‘2’) that changed to a fast increase of d(ΔF/F_0_)/dt at 6.4±0.3 ms after application of the electrical stimulus (marker ‘3’) and peaked at 1183±180 s^−1^ after 11.8±0.6 ms. In contrast the CT d(ΔF/F_0_)/dt signal did not reveal an obvious slow rising phase and did not start to rise until 2.2±0.3 ms after the first change of the SS signal (marker ‘2’) was observed. Maximal flux in the CT region was 3.5±0.3 times less than in the periphery and peaked 5.3±1.7 ms later. Fig. 5Bc shows regional (SS, CT) atrial Ca^2+^ fluxes in control and myocytes with disabled SR (TG+Caff). In the SS region d(ΔF/F_0_)/dt started to increase 2.1±0.1 ms (n = 6 atrial myocytes) after the electrical stimulus was applied and flux reached a maximum (164±30 s^−1^) after 6.5±0.5 ms, approximately at the time when in control cells SR Ca^2+^ release flux started. Ca^2+^ release flux in the CT region was 4.0±0.7 times smaller compared to the SS region.

**Figure 6:**
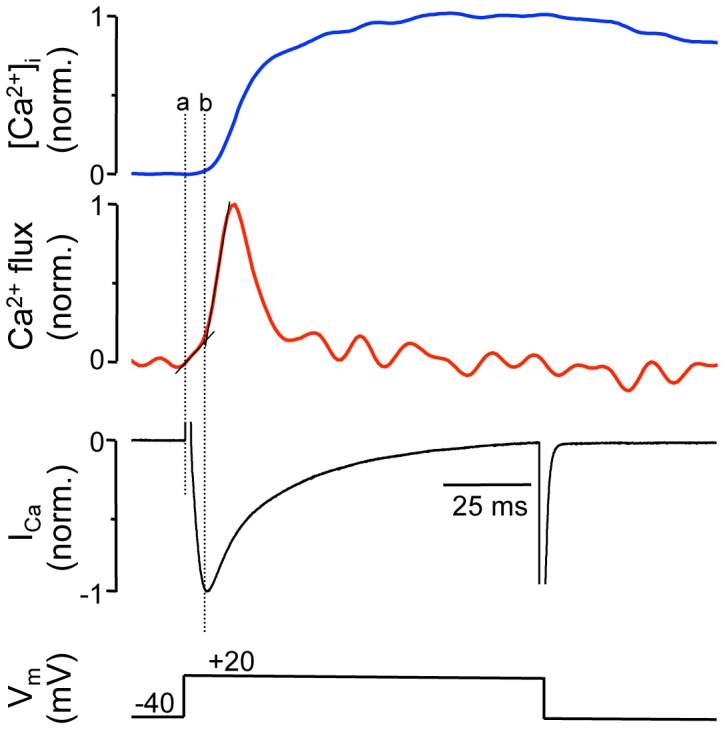
Simultaneous [Ca^2+^] and I_Ca_ measurements. Simultaneous measurements of [Ca^2+^]_i_ and I_Ca_ from a voltage-clamped (ruptured patch configuration) ventricular myocyte. Shown from top: [Ca^2+^]_i_ (ΔF/F_0_), Ca^2+^ flux (d(ΔF/F_0_)/dt), I_Ca_ and the command voltage. Traces represent averages of 15 successive depolarization pulses applied to the same cell and were normalized to their respective maximal amplitude. In these experiments whole-cell Fluo-4 fluorescence was recorded using a photomultiplier tube. Marker ‘a’: begin of depolarization pulse; marker ‘b’: change in flux rate, presumably identifying activation of CICR from the SR.

In summary, fast 2-D Ca^2+^ imaging enabled us to identify Ca^2+^ fluxes associated with Ca^2+^ entry and Ca^2+^ release from the SR. Next, we attempted to validate the optically measured Ca^2+^ entry and release signals (Fig. 5) with simultaneous measurements of [Ca^2+^]_i_ and membrane Ca^2+^ currents (I_Ca_) recorded with the patch clamp technique. Spatially averaged [Ca^2+^]_i_ and I_Ca_ measurements were obtained from single ventricular myocytes (Fig. 6). Cells where held at −40 mV and repetitively depolarized to +20 mV for 100 ms to elicit maximal LCCs. Fig. 6 shows from top to bottom, [Ca^2+^]_i_ (ΔF/F_0_), Ca^2+^ flux (d(ΔF/F_0_)/dt), I_Ca_ and the command voltage. The Ca^2+^ flux signal showed a slow steady increase shortly after the begin of the depolarization pulse (marker ‘a’). The slow increase of Ca^2+^ flux abruptly increased at marker ‘b’, giving way to a rapid rise of the Ca^2+^ flux rate. The distinct change in flux rate (marker ‘b’) occurred 5.7 ms after the begin of the depolarization pulse and correlated well with the begin of SR Ca^2+^ release determined by optical methods (marker ‘3’ in Fig. 5A).

In conclusion, the data presented in Figures 5 and 6 provide evidence that fast 2-D confocal Ca^2+^ imaging with sampling rates in the 1–2 kHz range can generate information on Ca^2+^ fluxes underlying ECC with a time resolution that is approaching the time resolution of the patch clamp technique, however has the advantage of lesser perturbation of the intracellular environment since the surface membrane of the cell remains intact.

## Discussion

In this study, we investigated the behavior and interactions of CRUs during electrically evoked Ca^2+^ transients and individual elementary Ca^2+^ release events (Ca^2+^ sparks) in atrial and ventricular myocytes with 2-D and line scan confocal imaging at high recording speeds. The key findings of our investigation were as follows:

First, the recruitment of individual CRUs by AP depolarization was spatially inhomogeneous and temporally asynchronous. Spatial inhomogeneity was particularly pronounced in atrial myocytes that lack a t-tubular membrane system. Second, high-speed confocal Ca^2+^ imaging allowed to resolve a distinct sequency of events during a Ca^2+^ transient, consisting of a latency period, Ca^2+^ entry and SR Ca^2+^ release, by an optophysiological approach [22], that has a time resolution comparable to electrophysiological recording (patch clamp) methods, however, is considerably less invasive since the integrity of cell membrane and cytosolic environment remains intact. Third, ultra-fast line scan recordings revealed step-like changes of [Ca^2+^]_i_ during the rising phase of Ca^2+^ sparks that may provide novel insights into RyR gating properties within individual CRUs.

Activation of SR Ca^2+^ release in atrial myocytes was asynchronous at two different levels. As has been described before [7], [10], [13], [26], [52], [53], due the lack (or poor development) of the t-tubular system, subsarcolemmal Ca^2+^ release originating at the peripheral couplings of the j-SR preceded Ca^2+^ release from the nj-SR in central regions of the cell, and peak CT [Ca^2+^]_i_ lagged behind by tens of milliseconds during AP-triggered Ca^2+^ transients (Fig. 1E). However, the initial activation of j-SR Ca^2+^ release in the cell periphery was also asynchronous and spatially inhomogeneous (Fig. 1B). Recruitment of detectable peripheral CRUs extended over at least 3 ms. A similar asynchronous activation pattern was found in ventricular myocyes where recruitment of all discernable CRUs required ∼4 ms. These numbers are low-end estimates. At later times the progressing global increase of [Ca^2+^]_i_ and Ca^2+^ diffusion away from activated release sites starts to obscure the recruitment of new sites and hampers their detectability at later times. Using line scan imaging numerous studies have found evidence for spatial inhomogeneities (e.g. [26], [48], [49]) that can be exacerbated in pathological conditions [50]. However, it is difficult to discern whether in line scan data apparent inhomogeneities arise from asynchronous activation of CRUs, silent or missing CRUs, or are the result of the inherent shortcoming of this recording mode that the scanned line potentially is missing CRUs (for example, when randomly placed between 2 neighboring Z-disks that are typically spaced ∼2 µm apart [13]). In 2-D imaging the problem is alleviated since the radial distance (i.e. the distance within the plane of a Z-disk) between CRUs is in the sub-micrometer range [29], [30], [32], [33], i.e. within the range of the spatial resolution of the microscope. In other words, upon AP stimulation all events recorded in 2-D mode originating from CRUs at the intersection of the focal plane and the plane of the Z-disk or from CRUs located on the Z-disk at distances that are within the point-spread function of the microscope (see Methods) will be detected. Consequently, every CRU that becomes activated during an AP within the resolved volume surrounding the focal plane will be captured. Thus, delays and asynchrony between activation of individual CRUs can more reliably be recorded.

Fast 2-D Ca^2+^ imaging allowed also to separate Ca^2+^ entry from SR Ca^2+^ release during an AP-induced Ca^2+^ transient by optical methods (Figures 5 and 6). By analyzing the first derivative of the Ca^2+^ transient three distinct phases could be discerned during the first ten milliseconds of the Ca^2+^ transient. First, a latency period of ∼2.5 ms between stimulus application and the first indication of an increase of [Ca^2+^]_i_ was observed. The second phase, characterized by a slow increase of [Ca^2+^]_i_ and lasting until 5.5 (ventricular) and 6.4 ms (atrial myocytes) after the begin of the pulse, represents Ca^2+^ entry across the surface membrane which was confirmed with simultaneous patch clamp and [Ca^2+^]_i_ measurements (Fig. 6). This conclusion was supported by the fact, that the rise of [Ca^2+^]_i_ was still observed when SR Ca^2+^ release was disabled, and the change in [Ca^2+^]_i_ coincided well with the current recordings. The third phase, characterized by an abrupt increase of release flux by approximately an order of magnitude, peaked 10–12 ms after the pulse. Phase 3 was completely abolished when the SR was disabled. The data indicate that 2-D Ca^2+^ imaging with 1–2 kHz time resolution is capable of distinguishing between trigger and released Ca^2+^ in intact myocytes by an optophysiological approach, with the advantage that cell membrane and cytosolic environment are kept undisturbed.

Despite substantial progress made towards the understanding of Ca^2+^ release and ECC in atrial tissue, several key questions have remained unanswered. For example, with the lack of t-tubules the CRUs of the nj-SR (which account for a large majority of atrial CRUs) do not have the characteristics of a typical couplon. Specifically, the RyRs are not facing into a narrow diadic cleft where upon activation of LCCs [Ca^2+^]_i_ rapidly increases to concentrations that are several orders magnitude higher than resting [Ca^2+^]_i_. With the inherent low Ca^2+^-sensitivity of the RyR [54], [55] it is difficult to reconcile how [Ca^2+^]_i_ can rise sufficiently high in the vicinity of RyRs of the nj-SR to lead to channel opening and Ca^2+^ release. Furthermore, it has even been suggested that Ca^2+^ sequestering mitochondria in the subsarcolemmal space of atrial myocytes constitute a Ca^2+^ barrier [15], making the propagation of CICR from peripheral j-SR to nj-SR even more problematic. As shown previously [41], atrial sparks have a relatively large Ca^2+^ flux (∼11 pA) and large numbers of channels involved (20–30 RyRs) with an enhanced propensity to open and a diminished influence of negative feedback mechanisms as well as an extended spatial spread (see also [40]). All these features tend to facilitate propagation of release from nj-SR. Interestingly, in contrast to Ca^2+^ sparks recorded from nj-SR and sparks in ventricular cells, the location of maximal fluorescence identifying a point source of Ca^2+^ release of peripheral atrial sparks is not spatially fixed, but moves in centripetal direction during the evolution of the spark (Fig. 2C). Thus, peripheral atrial sparks reveal some inherent feature for directional propagation that facilitates the saltatory advancement of activation from j-SR to the nj-SR, although it remains to be determined what constitutes the structural or functional basis of this special feature.

The question how many RyRs are found in an individual CRUs has remained rather elusive. Earlier reports suggested the number to be in the range of 50–250 RyRs [23], [29], [30], [31], [32], [33], [35]. However, novel advanced technologies, including super-resolution imaging techniques [56] that go beyond diffraction-limited resolution, and 3-dimensional electron microscopy tomography [57], have revealed new aspects of the make up of the cardiac CRU [36], [58], [59]: CRUs are variable in size, of irregular geometry, lack the regular arrays of RyRs typical of skeletal muscle, and show a smaller density of RyRs than previously assumed. Furthermore, individual CRUs can assemble in groups to form ‘super-clusters’ resulting in variable degrees of coordination of Ca^2+^ release from sub-clusters. These recent studies that generated evidence of incomplete packaging of CRUs with RyRs and the existence of sub-domains of smaller sizes have corrected the number of RyRs per CRU towards lower values in the range of a few tens to less than ten RyRs [29], [34], [36]. While recent technological advances for studying CRUs have generated valuable novel insights, it is still far from being established how many RyRs actively contribute to Ca^2+^ release of a spark. The estimates, just for cardiac myocytes alone, range from possibly only one or only a very small fraction of the cluster (<10 RyRs), to whole-cluster activation with possibly contribution from several hundred channels [21], [32], [37], [38], [39], [40], [41], [42]. The ionic current or ionic flux underlying a spark is estimated to be in the range of 3–5 pA [38], [40], [60], [61], [62], [63], although it tends to be higher in atrial myocytes (11 pA; [41]). Ca^2+^ currents through single cardiac RyR channels reconstituted in lipid bilayer and recorded under physiological ionic conditions gave unitary current values of 0.3–0.7 pA [64]. Based on these estimates only ∼5–35 RyRs passing current simultaneously would participate in a single spark. This leads to the paradox that the estimated number of participating channels is nearly an order of magnitude smaller than the higher estimates for the number of RyRs per CRU. Recent results indicate that the current underlying a spark is quantal in nature. By using ‘sparklets’ - local Ca^2+^ signals of known magnitude arising from LCCs [39] - to calibrate Ca^2+^ sparks, it could be demonstrated that Ca^2+^ sparks have a quantal substructure and that Ca^2+^ spark release flux is made up of quanta of 1.2 pA. Furthermore, the number of quanta in a spark varies dynamically from one to ≤8 [62]. Whether single quanta reflect the opening of a single RyR or a group of tightly coupled channels is unclear. Cooperativity of activation and inactivation among release channels or ‘coupled gating’ [65], [66], [67] could enable entire groups of channel to generate a single quantum. In support of multichannel quanta is also the observation that the measured quantum size (1.2 pA) is larger than the estimates for RyR unitary currents (∼0.5 pA, lasting ∼10 ms) under physiological ionic conditions. Thus, quantal release with a low number of individual quanta does not necessarily rule out the involvement of a larger number of RyRs. In addition, the observation of RyR super-clusters raises the possibility that quantal release reflects the concerted activation of sub-clusters [56]. Since sub-clusters vary in size and number of RyRs this model could explain quantal release with quanta of variable sizes, however this would require a looser definition of the term ‘quantal’ to avoid violation of a stricter definition of ‘quanta’ which suggests entities of identical magnitude. Our data shown in Fig. 4 may point towards coordinated gating of an entire RyR cluster with a high degree of cooperativity. The d(ΔF/F_0_)/dt signal shown in Fig. 4B, at first glance, is reminiscent of single-channel gating with the peaks indicating channel openings. On the other hand, we have shown previously that in the same species atrial Ca^2+^ sparks involve release from 20–30 RyRs [41]. Coupled gating with a high degree of cooperativity within a CRU, where the entire RyR cluster behaves essentially as a single channel, could reconcile this apparent paradox. Upon closer examination, in the d(ΔF/F_0_)/dt signal in Fig. 4B at least three distinct amplitude levels can be identified (grey bars). Thus, the data could be consistent with coupled gating of the entire cluster or a small number of subclusters, but do not exclude a quantal nature of Ca^2+^ release since quantal release consisting of a small number of quanta (even 1 or 2 quanta only) has been demonstrated [62].

While entirely speculative at this point, the progressive activation of RyR sub-clusters within a CRU may also explain the centripetal propagation of Ca^2+^ release found in subsarcolemmal atrial CRUs (Fig. 2C). Furthermore, to reconcile the paradox of a presumably small fraction of active channels and a large number channels present in a CRU, several possibilities need to be considered. For one, the unitary RyR current could be much smaller (possibly in the femto-ampere range) during a spark than measured in the bilayer because the rapid rise of Ca^2+^ in the diadic cleft together with local SR depletion could significantly decrease the driving force for Ca^2+^ flux. Conceivably, powerful, however yet to be identified inhibitory mechanisms, negative cooperativity among RyRs (opening of a RyR reduces the open probability of its neighbors), strong Ca^2+^-dependent RyR inactivation or the recently proposed ‘pernicious attrition’ Ca^2+^ release termination mechanism [68] could prevent the activation of an entire CRU by intra-cluster CICR. As recently suggested, Mg^2+^ binding to the RyR could be responsible for such a strong inhibitory mechanism [69].

In summary, we demonstrate here that high-speed confocal imaging with sampling rates in the 1–40 kHz range in conjunction with high quantum yield fluorescent indicators allows to study Ca^2+^ dynamics and intricacies of single SR Ca^2+^ release units. With this technique we showed that dynamics of Ca^2+^ entry and CICR can reliably be distinguished at the level of individual CRUs in intact cells, that Ca^2+^ sparks of atrial myocytes have inherent properties that facilitate centripetal propagation of activation, and that the rising phase of Ca^2+^ sparks reveals step-like kinetics which bears potentially valuable information about intra-CRU channel gating. These findings have important ramifications for the understanding of the structural and functional organization of CRUs, CICR and ECC.
